# Identification of Methicillin-Resistant Staphylococcus Aureus From Methicillin-Sensitive Staphylococcus Aureus and Molecular Characterization in Quanzhou, China

**DOI:** 10.3389/fcell.2021.629681

**Published:** 2021-01-21

**Authors:** Zhimin Bai, Min Chen, Qiaofa Lin, Ying Ye, Hongmei Fan, Kaizhen Wen, Jianxing Zeng, Donghong Huang, Wenfei Mo, Ying Lei, Zhijun Liao

**Affiliations:** ^1^Department of Biochemistry and Molecular Biology, School of Basic Medical Sciences, Fujian Medical University, Fuzhou, China; ^2^Department of Clinical Laboratory, Jinjiang Municipal Hospital, Jinjiang, China; ^3^Microbiological Laboratory Sanming Center for Disease Control and Prevention, Sanming, China; ^4^Department of Clinical Laboratory, The Second Affiliated Hospital of Fujian Medical University, Quanzhou, China; ^5^Department of Clinical Laboratory, Quanzhou Women’s and Children’s Hospital, Quanzhou, China

**Keywords:** Staphylococcus aureus, feature vector, virulence factor, molecular characterization, antibiotic resistance, machine learning

## Abstract

To distinguish Methicillin-Resistant Staphylococcus aureus (MRSA) from Methicillin-Sensitive Staphylococcus aureus (MSSA) in the protein sequences level, test the susceptibility to antibiotic of all Staphylococcus aureus isolates from Quanzhou hospitals, define the virulence factor and molecular characteristics of the MRSA isolates. MRSA and MSSA Pfam protein sequences were used to extract feature vectors of 188D, n-gram and 400D. Weka software was applied to classify the two Staphylococcus aureus and performance effect was evaluated. Antibiotic susceptibility testing of the 81 Staphylococcus aureus was performed by the Mérieux Microbial Analysis Instrument. The 65 MRSA isolates were characterized by Panton-Valentine leukocidin (*PVL*), X polymorphic region of Protein A (*spa*), multilocus sequence typing test (*MLST*), staphylococcus chromosomal cassette mec (*SCC*mec) typing. After comparing the results of Weka six classifiers, the highest correctly classified rates were 91.94, 70.16, and 62.90% from 188D, n-gram and 400D, respectively. Antimicrobial susceptibility test of the 81 Staphylococcus aureus: Penicillin-resistant rate was 100%. No resistance to teicoplanin, linezolid, and vancomycin. The resistance rate of the MRSA isolates to clindamycin, erythromycin and tetracycline was higher than that of the MSSAs. Among the 65 MRSA isolates, the positive rate of *PVL* gene was 47.7% (31/65). Seventeen sequence types (*STs*) were identified among the 65 isolates, and *ST59* was the most prevalent. *SCCmec* type III and IV were observed at 24.6 and 72.3%, respectively. Two isolates did not be typed. Twenty-one *spa* types were identified, *spa t*437 (34/65, 52.3%) was the most predominant type. MRSA major clone type of molecular typing was *CC59-ST59-spa t437-IV* (28/65, 43.1%). Overall, 188D feature vectors can be applied to successfully distinguish MRSA from MSSA. In Quanzhou, the detection rate of *PVL* virulence factor was high, suggesting a high pathogenic risk of MRSA infection. The cross-infection of CA-MRSA and HA-MRSA was presented, the molecular characteristics were increasingly blurred, HA-MRSA with typical CA-MRSA molecular characteristics has become an important cause of healthcare-related infections. *CC59-ST59-spa t437-IV* was the main clone type in Quanzhou, which was rare in other parts of mainland China.

## Introduction

Staphylococcus aureus has been considered the mainly pathogen that cause skin and soft-tissue infections, central nervous system infections, necrotizing pneumonia and infections associated with intravascular devices (Conceicao et al., 2007; Nadig et al., 2010; Rosa et al., 2016). Staphylococcus aureus is categorized into two groups, methicillin-sensitive Staphylococcus aureus (MSSA) and methicillin-resistant Staphylococcus aureus (MRSA) (Ahmed and Mukherjee, 2018). Which is based on the well-known differences of the *mec*A gene conferred to the pathogen, and the significant difference of the biofilm formation between MRSA and MSSA strains (Gidari et al., 2020). MRSA is responsible for most global Staphylococcus aureus bacteremia cases, and MRSA infection is related to poorer clinical outcomes than MSSA (Hassoun et al., 2017). Thus, MRSA is an important nosocomial pathogen that is being observed with increasing frequency in community settings. However, some studies have shown that tst-positive MSSA strains belonging to *ST1*, *ST8*, and *ST30* are a potential source of tst-positive community-acquired MRSA and speculated that the tst-positive MRSA clones may have emerged from their respective MSSA counterparts. Therefore, MRSA and MSSA may owe the tst gene as an aid to targeted infection control (Schlebusch et al., 2009). Machine learning methods have a broad application in the bioinformatics, especially in the biological classification fields (Jiang et al., 2013; Liao et al., 2017, 2018a; Xu et al., 2018, 2019; Yu et al., 2018, 2020a,b; Ding et al., 2019; Liu G. et al., 2019; Liu B. et al., 2020; Shen et al., 2019a,b; Li et al., 2020; Shao et al., 2020; Wang H. et al., 2020; Zhao et al., 2020a,b). Here, Machine learning algorithm was performed to accomplish the classification of MRSA and MSSA based on their protein sequences (Liao et al., 2018b).

Since the first methicillin-resistant staphylococcus aureus (MRSA) reported in 1961 (Jevons, 1961), MRSA isolates were soon recovered from other European countries, and later from the United States, Japan, and Australia. At the same time, the resistance rate of MRSA was so high that it gave rise to significant morbidity and mortality. Currently, MRSA is also resistant to various non-β-lactam antibiotics, such as erythromycin, clindamycin, gentamicin, ciprofloxacin, and levofloxacin (Jiun-Ling et al., 2010). MRSA has caused an increasing public and occupational health concern.

In the early 1990s, community-associated MRSA (CA-MRSA) first broke out sporadically in several parts of Western Australia and the United States (Udo et al., 1993; Diekema et al., 2014). In 1999, The Centers for Disease Control and Prevention (CDC) reported that four children from Minnesotans and North Dakota died of sepsis in CA-MRSA infection, causing widespread concern (CDC, 1999). CA-MRSA is different from hospital-acquired MRSA (HA-MRSA) and has its own unique characteristics in virulence factors, genetic characteristics, epidemiology, and clinical manifestations. CA-MRSA infection most commonly affects skin and soft tissues, it is also associated with severe invasive diseases such as necrotizing pneumonia and sepsis, which often infect healthy young people such as students, athletes, and military personnel. The outbreak of CA-MRSA is associated with several common features, including close contact, poor sanitation, sharing among public goods or public facilities, skin surface abrasions, and lack of medical care to treat infections. Generally, CA-MRSA carries *PVL* virulence factors belonging to type IV *SCCmec*, and its *SCCmec* elements are relatively small, which is conducive to widespread transmission. Therefore, it is easy to form a wide range of epidemics. Usually without carrying other antibiotic resistance genes and therefore non β-lactam antibiotics are sensitive. While HA-MRSA usually contains large *SCCmec* such as type I, type II or type III, and contains a variety of anti-drug genes, the resistance of HA-MRSA isolates is not limited to β-lactam antibiotics. Pathogens are often resistant to multiple antibiotics.

The prevalence and resistance phenotypes of MRSA in different countries and regions are different and always changing over time. After reviewing the literature of nearly a decade, almost no paper reported on the molecular epidemiological investigation of MRSA in Quanzhou. This study analyzed the antimicrobial resistance of the Staphylococcus aureus isolated from several Three-A hospitals in Quanzhou, and tested the *PVL* virulence factor, *spa* typing, *MLST* typing and *SCCmec* typing of MRSA in this region, which provided a reference for clinical treatment of MRSA infection and response to explosive epidemics.

## Materials and Methods

### Data Retrieval and Treatment

All the primary sequences of both MRSA and MSSA Pfam proteins (in FASTA files) were retrieved from the UniProt database^1^, the raw data are preprocessed by cd-hit program^2^ to merge the sequence similarities and reduce the complexity. To avoid bias in the classifier, we set the identity at rigorous 30% similarity and remove the intersecting sequences, finally we obtained the results of 439 MRSA sequences as positive dataset and 62 MSSA entries as negative dataset. Since the MRSA sequences are seven times that of the MSSA sequences, the MRSA sequence is divided into seven, and the positive sequence and the negative sequence 1:1 constitute seven sets of data.

### Construction of Feature Vectors for Positive and Negative Sequences

Feature selection (Wang G. et al., 2008; Zhao et al., 2015; Cheng and Hu, 2018; Du et al., 2018; Su et al., 2018; Tang et al., 2018; Wei et al., 2018a,b; Cheng et al., 2020) is the important process to select the extracted features that give the best classification results. To predict the potential MRSA from MSSA at the amino acid sequence level, firstly, we extracted the feature vectors from positive versus negative protein sequence dataset by using three novel machine-learning-based methods developed by our group, that are 188D, n-gram and 400D feature vectors (Wang G. et al., 2010; Liao et al., 2016; Liu and Jiang, 2016; Xinrui et al., 2018; Leyi et al., 2019; Liu B. et al., 2019; Yu et al., 2019; Zhang and Liu, 2019; Ao et al., 2020b).

### Construction of Classifier With Weka and Classification Evaluation

**FIGURE 1:**
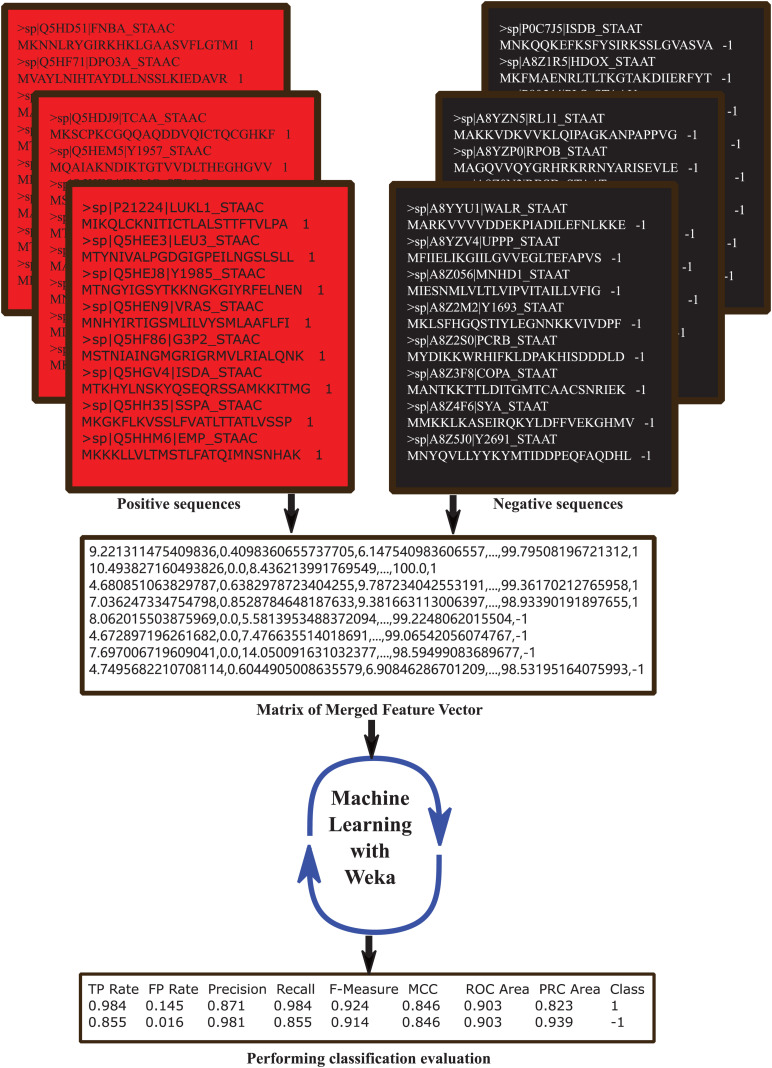
The computational framework of classification prediction for distinguishing MRSA from MSSA based on amino acid sequences. Firstly, both MRSA (positive) and MSSA (negative) protein sequences were retrieved from UniProt and pretreated by CD-HIT (30% similarity) and obtained dataset containing 439 MRSA and 62 MSSA entries. Secondly, MRSA were randomly divided into 7 groups with 62 entries in each group, each group MRSA and MSSA were extracted the feature vectors including 188D, n-gram and 400D methods. Thirdly, positive and negative feature vectors matrix were imported into Weka (10-fold cross-validation) and the six best performing classifiers were selected for further analysis. Finally, the 4 common measurements (Sn, Sp, Acc, and MCC) were used to evaluate classification performance.

Weka^3^ is a machine learning software for many applications that is widely used for teaching and research (Ye et al., 2019), and the Classify module contains several kinds of classifiers such as bayes, functions, lazy, meta, misc, rules, and trees in Weka Explorer. We use all the classifiers to train and select the best 6 performed ones to compare each other: AdaBoostM1, RandomSubSpace, DecisionTable, OneR, RandomForest, and REPTree. All the classifiers were set the parameters as default and test mode set as 10-fold cross validation. The identification process was showed in Figure 1.

We use four common measurements to illuminate the performance quality, that is Sensitivity (Sn), Specificity (Sp), Accuracy (Acc) and Matthew’s correlation coefficient (MCC)were adopted to evaluate the above three methods and four classifiers. These methods are formulated as follows (Wei et al., 2014, 2017a,b, 2018c; Zhao et al., 2017; Wang G. et al., 2018; Cheng, 2019; Cheng et al., 2019; Yang et al., 2019; Ao et al., 2020a; Hasan et al., 2020; Qiang et al., 2020; Tang et al., 2020):

$$S\,n=\frac{T\,P}{\text{TP}+\text{FN}}$$

$$S\,p=\frac{T\,N}{\text{TN}+\text{FP}}$$

$$A\,c\,c=\frac{T\,P+T\,N}{T\,P+F\,P+T\,N+F\,N}$$

$$M\,C\,C=\frac{T\,P*T\,N-F\,P*F\,N}{\sqrt{\left(T\,P+F\,N\right)\,\left(T\,P+F\,P\right)\,\left(T\,N+F\,P\right)\,\left(T\,N+F\,N\right)}}$$

Where *TP*, *TN*, *FP*, and *FN* stand for the numbers of true positive, true negative, false positive, and false negative, respectively.

### Clinical Strains

A total of 81 non-repetitive Staphylococcus aureus strains were isolated from the outpatients and inpatients in three tertiary hospitals, which were Second Affiliated Hospital of Fujian Medical University, Quanzhou Children’s Hospital and Jinjiang Municipal Hospital, between October 2018 and July 2019. Staphylococcus aureus was identified by the Mérieux automated bacterial tester. According to the Clinical and Laboratory Standards Institute (CLSI) Antibiotic Sensitivity Test Execution Standard, Methicillin-Resistant Staphylococcus aureus (MRSA) was the MIC value of oxacillin ≥4 μg/ml and the plasma coagulase was also positive. At the same time, the *mec*A gene was detected by PCR to confirm. All the isolates were stored at −80°C for further experiments.

### Ethics Statement

After inquiring the hospital, this study didn’t require any ethics statement because no work was developed with human samples. Strains were isolated directly from the patients to plates. Strains were collected not only for this study, but also for diagnosing of infection. Patient identifying information was collected by medical doctors as part of the routine hospital patient care procedure, and a number was assigned to each patient. Information arrived at the laboratory with this number after isolating and identifying all strains. Patient consents for collecting their clinical signs, medical histories, and characteristics were obtained during the admission of the hospital as a part of the routine hospital patient care procedure.

### Antimicrobial Susceptibility Testing of the 81 Staphylococcus Aureus Strains

The antimicrobial susceptibility testing was conducted on all Staphylococcus aureus strains by the Mérieux Microbial Analysis Instrument according to the guidelines of CLSI M100-S29. The antibiotics tested were penicillin, linezolid, teicoplanin, vancomycin, ciprofloxacin, gentamicin, levofloxacin, clindamycin, Sulfamethoxazole/trimethoprim, erythromycin, rifampicin, and tetracycline. Staphylococcus aureus ATCC25923 and ATCC29213 were used for quality control.

### *PVL* Gene and Molecular Typing of the 65 MRSA Isolates

#### Extraction of Genomic DNA

Sixty-five MRSA clinical isolates and standard strains were inoculated on blood agar culture plates overnight for 16–18 h, and DNA was extracted according to the bacterial genomic DNA rapid extraction kit. The obtained DNA was dissolved in 50 μl of TE Buffer and placed in an autoclaved eppendorf tube, and stored at −20°C.

#### Detection of *PVL* Gene

The *PVL* gene was amplified by PCR as described previously (Mcclure et al., 2006). The amplified product was performed to agarose gel electrophoresis. One amplified band appeared at 433 bp as the *PVL* gene, and 146 bp was the *mecA* gene. The identity of the PCR product was confirmed by sequencing. *PVL* quality control strain was CCUG46923.

#### *Spa* Typing

*Spa* typing was performed as described previously (Harmsen et al., 2003). Purified *spa* PCR products were sequenced, and short sequence repeats were assigned by using the *spa* database website^4^. The *spa* complex was defined by visual analysis, whereby *spa* types with similar short sequence repeats were clustered into the complexes previously described by Ruppitsch et al. (2006).

#### *SCCmec* Typing

The *SCCmec* types were determined by a multiplex PCR developed by Oliveira et al. (2006). Non-types (*NT*) were defined as isolates showing unexpected fragments or lacking some fragments as determined by multiplex PCR. The quality controls were MRSA NCTC10442 (*SCCmec* I), MRSA N315 (*SCCmec* II), MRSA 85/2082 (*SCCmec* III), MRSA JCSC 4744 (*SCCmec* IV).

#### MLST and Data Analysis

Multilocus sequence typing test was carried out as described previously (Enright et al., 2000). The sequences of the PCR products were compared with the existing sequences available on the MLST website^5^ for Staphylococcus aureus, and the allelic number was determined for each sequence. The clustering of related *STs*, which were defined as clonal complexes (CCs), was determined by using the program eBURST (based upon related sequence types) (Feil et al., 2004).

## Results

### Classification of Positive and Negative Proteins

**FIGURE 2:**
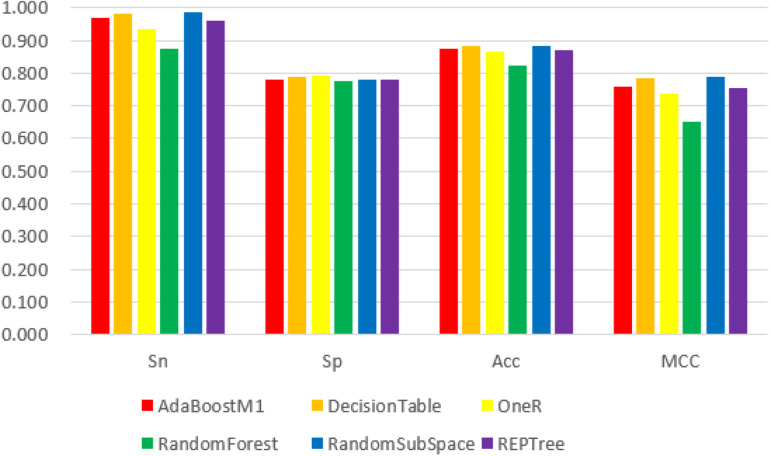
The 6 classifiers performance comparison using Sensitivity (Sn), Specificity (Sp), Accuracy (Acc) and Matthew’s Correlation Coefficient (MCC) values for 188D features. Among them, the RandomSubSpace classifier performs best in the four evaluation indexes with its values 98.8, 78.2, 88.5, and 78.8%, respectively.

**FIGURE 3:**
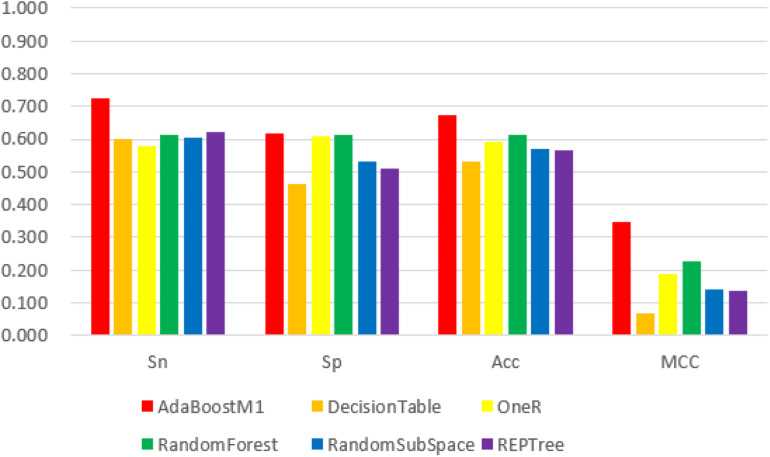
The 6 classifiers performance comparison using Sensitivity (Sn), Specificity (Sp), Accuracy (Acc) and Matthew’s Correlation Coefficient (MCC) values for n-gram features. Among them, the AdaBoostM1 classifier performs best in the four evaluation indexes with its values 72.6, 61.7, 67.1, and 34.5%, respectively. However, all evaluation index values of this feature are lower than 188D.

**FIGURE 4:**
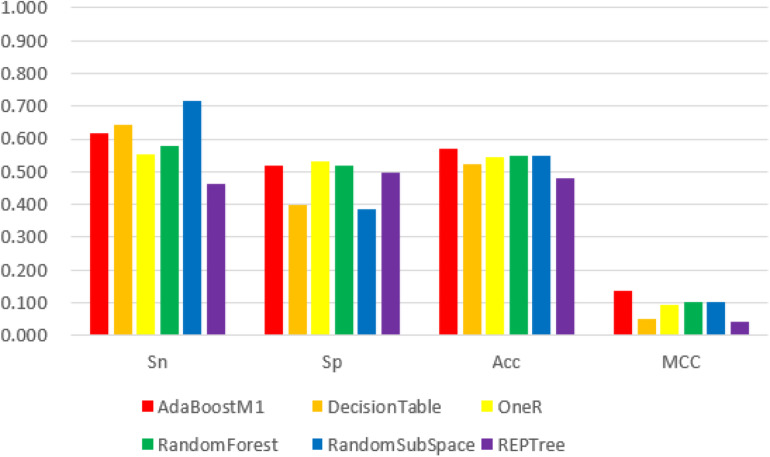
The 6 classifiers performance comparison using Sensitivity (Sn), Specificity (Sp), Accuracy (Acc) and Matthew’s Correlation Coefficient (MCC) values for 400D features. For the Sn index, RandomSupSpace classifier performs best with a value 71.8%, and OneR classifier performs best with a value 53.2% for Sp index, but for the Acc and MCC indexes the AdaBoostM1 classifier performs best with its value 56.9%, 13.8%, respectively. Among the above three features, 400D get the worst performance.

We obtained the 188D, n-gram and 400D feature vector datasets from both positive and negative groups and used them as input to the Weka explorer. The results showed that the highest correctly classified rates were 91.94, 70.16, and 62.90%, respectively. The four common classification measurement values from 188D, n-gram and 400D feature vectors are illustrated in Figures 2–4.

### Identification of MRSA/MSSA and Antimicrobial Resistances

Of the 81 strains of Staphylococcus aureus, 65 were MRSA, 16 strains were MSSA strains. According to the definition of HA-MRSA and CA-MRSA, 65 strains of Staphylococcus aureus were divided into 22 HA-MRSAs and 43 CA-MRSAs.

**FIGURE 5:**
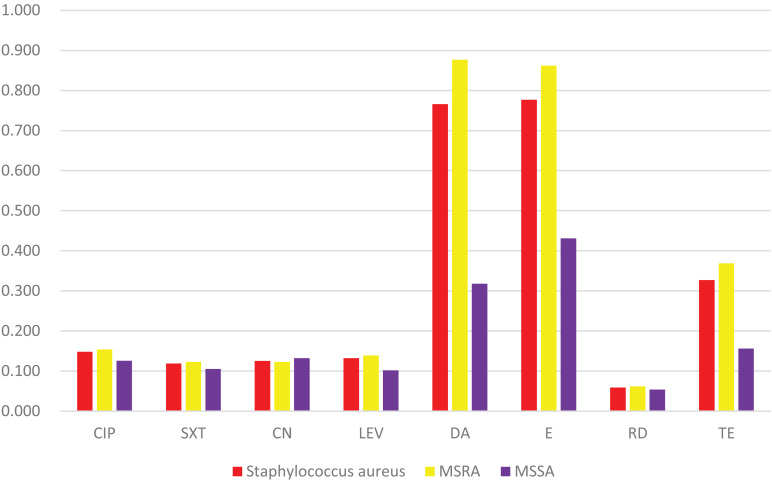
Resistance rates of eight antibiotics. It shows that the MRSA isolates possess significantly higher resistance rates to DA, E, and TE than the MSSA isolates. CIP (ciprofloxacin), SXT (Sulfamethoxazole/trimethoprim), CN (gentamicin), LEV (levofloxacin), DA (clindamycin), E (erythromycin), RD (rifampicin), TE (tetracycline).

Penicillin-resistant rate was 100%. No resistance to teicoplanin, linezolid, and vancomycin. The antimicrobial resistance rates to ciprofloxacin, sulfamethoxazole/trimethoprim, gentamicin, levofloxacin, clindamycin, erythromycin, rifampin, and tetracycline were 14.8, 11.9, 12.5, 13.2, 76.6, 77.7, 5.9, and 32.7%, respectively. Statistical analysis show that the MRSA isolates had significantly higher resistance rates to clindamycin than the MSSA isolates (87.7% vs. 31.8%, *p* < 0.001), erythromycin (86.2% vs. 43.1%, *p* < 0.001), tetracycline (36.9% vs. 15.6%, *p* = 0.03). The multi-resistance rate of MRSA was 93.8% (61/65) (Figure 5).

### *PVL* Gene Screening

**FIGURE 6:**
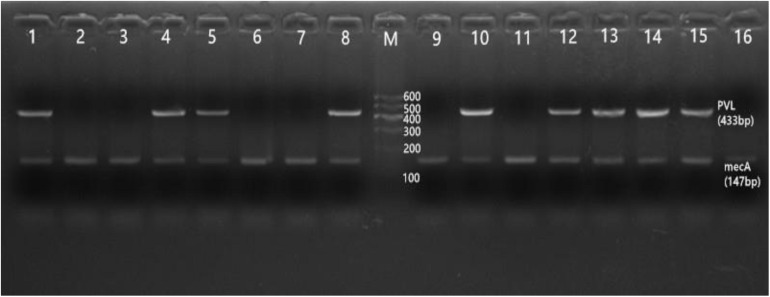
Results of agarose gel electrophoresis. This figure shows that all MRSA isolates can be detected with *mecA* gene, as well as the positive and negative test results of the *PVL* gene. M: 600 bp DNA Ladder; 1: positive *PVL* control; 2: *PVL* negative control. 4, 5, 8, 10, 12–15: *PVL* positive strains; 3, 6, 7, 9, 11, 16: *PVL* negative strains. 1–16: *mecA* gene.

Of the 65 MRSA isolates, thirty-one were *PVL* positive (31/65, 47.7%), among which nine were detected by HA-MRSA (9/22, 40.9%), and twenty-two were detected by CA-MRSA (22/43, 51.2%) (*p* > 0.5). The *mec*A gene was detected in all MRSA (Figure 6).

### Molecular Typing

Sixteen isolates belonged to *SCCmec* type III and forty-seven belonged to *SCCmec* type IV. Two MRSA isolates could not be *SCCmec* typed. Twenty-one *spa* types were identified. T437 (34/65, 52.3%) was the most common, followed by t030 (6/65, 9.2%), t062 (3/65, 4.6%), t309 (3/65, 4.6%), and t13774 (2/65, 3.1%).

Among all MRSA isolates, sixteen sequence types (STs) were identified by MLST. The most common *ST* was *ST59* (36/65, 55.4%), followed by *ST239* (6/65, 9.2%) and *ST5* (4/65, 6.2%). Ten clonal complexes (*CCs*) were identified by eBURST. *CC59* (37/65, 56.9%) was the most common clone, followed by *CC8* (6/65, 9.2%) and *CC5* (4/65, 6.1%) (Table 1). MRSA major clone types of molecular typing were *CC59-ST59-spa* t437-IV (28/65, 43.1%), followed by *CC59-ST59-spa* t437-III (6/65, 9.2%), *CC8-ST239-spa* t030-III/IV (6/65, 9.2%), and *CC5-ST5-spa* t062-IV (4/65, 6.1%), respectively (Table 1).

**TABLE 1:** *MLST, spa, SCCmec, PVL, mecA*, and CA/HA-MRSA of the 65 MRSA isolates.

	***CC***	***MLST***	***spa***	***SCCmec***	***PVL***	***mecA***	**CA/HA-MRSA**
1	CC8	ST239	t030	IV	−	+	HA-MRSA
2	CC59	ST59	t437	IV	+	+	HA-MRSA
3	CC45	ST508	t529	IV	−	+	CA-MRSA
4	CC30	ST30	t019	IV	+	+	HA-MRSA
5	CC59	ST59	t437	IV	−	+	CA-MRSA
6	CC1	ST944	t3590	IV	+	+	CA-MRSA
7	CC8	ST239	t030	III	−	+	HA-MRSA
8	CC59	ST59	t437	III	−	+	HA-MRSA
9	CC88	ST88	t13774	III	−	+	CA-MRSA
10	CC8	ST239	t030	IV	−	+	HA-MRSA
11	CC59	ST59	t1894	III	+	+	CA-MRSA
12	CC59	ST59	t437	IV	+	+	HA-MRSA
13	CC59	ST59	t437	IV	+	+	CA-MRSA
14	CC88	ST88	t13774	IV	−	+	CA-MRSA
15	CC59	ST59	t437	IV	+	+	CA-MRSA
16	CC59	ST59	t437	IV	−	+	CA-MRSA
17	CC59	ST338	t441	IV	+	+	CA-MRSA
18	CC59	ST59	t437	IV	−	+	CA-MRSA
19	CC59	ST59	t437	IV	+	+	CA-MRSA
20	CC8	ST239	t030	IV	−	+	CA-MRSA
21	CC59	ST59	t437	IV	−	+	HA-MRSA
22	CC5	ST5	t062	IV	−	+	CA-MRSA
23	CC59	ST59	t437	III	+	+	CA-MRSA
24	CC59	ST59	t437	IV	−	+	CA-MRSA
25	CC59	ST59	t437	IV	+	+	CA-MRSA
26	CC45	ST45	t16027	IV	−	+	CA-MRSA
27	CC59	ST59	t437	IV	+	+	CA-MRSA
28	CC59	ST59	t437	IV	+	+	CA-MRSA
29	CC30	ST31	t5351	NT	+	+	HA-MRSA
30	CC59	ST59	t437	IV	+	+	CA-MRSA
31	CC59	ST59	t437	IV	−	+	CA-MRSA
32	CC59	ST59	t437	IV	−	+	CA-MRSA
33	CC59	ST59	t437	IV	+	+	CA-MRSA
34	CC59	ST59	t437	III	+	+	HA-MRSA
35	CC15	ST18	t624	NT	+	+	CA-MRSA
36	CC22	ST22	t309	III	+	+	CA-MRSA
37	CC59	ST59	t437	III	−	+	CA-MRSA
38	CC30	ST72	t2383	IV	−	+	CA-MRSA
39	CC59	ST59	t437	III	+	+	CA-MRSA
40	CC59	ST59	t437	IV	+	+	CA-MRSA
41	CC8	ST239	t030	III	−	+	HA-MRSA
42	CC22	ST22	t309	III	+	+	HA-MRSA
43	CC59	ST59	t437	IV	−	+	HA-MRSA
44	CC59	ST59	t437	IV	+	+	CA-MRSA
45	CC30	ST398	t034	IV	+	+	HA-MRSA
46	CC59	ST59	t437	IV	+	+	HA-MRSA
47	CC59	ST59	t437	IV	+	+	HA-MRSA
48	CC1	ST94	t779	IV	−	+	CA-MRSA
49	CC5	ST5	t062	IV	−	+	CA-MRSA
50	CC59	ST59	t437	IV	−	+	CA-MRSA
51	CC1	ST1	t127	IV	−	+	CA-MRSA
52	CC59	ST59	t437	IV	−	+	CA-MRSA
53	CC5	ST5	t172	IV	−	+	HA-MRSA
54	CC59	ST59	t437	IV	−	+	HA-MRSA
55	CC8	ST239	t030	III	−	+	HA-MRSA
56	CC5	ST5	t062	IV	−	+	HA-MRSA
57	CC59	ST59	t437	IV	+	+	CA-MRSA
58	CC59	ST59	t437	IV	−	+	HA-MRSA
59	CC59	ST59	t437	III	+	+	CA-MRSA
60	CC59	ST59	t1751	III	−	+	HA-MRSA
61	CC59	ST59	t437	IV	+	+	CA-MRSA
62	CC22	ST22	t309	III	+	+	CA-MRSA
63	CC45	ST45	t015	IV	−	+	CA-MRSA
64	CC15	ST15	t2613	III	−	+	CA-MRSA
65	CC5	ST5	t062	IV	+	+	CA-MRSA

*CC, clonal complex; *ST*, sequence type; *NT*, not typing.*

### Comparison of Antimicrobial Resistance Rates Between *CC59-ST59-spa* t437-IV and Other Types of MRSA

Comparing the antibiotic resistance rate between *CC59-ST59-spa* t437-IV clone and other types, this study found that the resistance rate of *CC59-ST59-spa* t437-IV clone to *CIP* (ciprofloxacin), *CN* (gentamicin) and *RD* (rifampicin) was lower than other clone types (*p* < 0.05) (Table 2).

**TABLE 2:** Comparison of resistance rates between *CC59-spa* t437 clones and other types.

	**ST59-t437**	**Non- ST59-t437**	**χ^2^-value**	***P*-value**
	**(*n* = 34), R^*a*^(%)**	**(*n* = 31), R^*a*^(%)**		
*CIP*	2.9	29.1	8.48	0.004
*SXT*	26	16.1	1.508	0.22
*CN*	2.9	22.6	5.795	0.016
*LEV*	5.9	22.6	3.79	0.052
*DA*	88.2	87.1	0.019	0.889
*E*	88.2	83.9	0.259	0.611
*RD*	0	12.9	4.675	0.031
*TE*	35.3	38.7	0.081	0.776

*CIP, ciprofloxacin; *SXT*, Sulfamethoxazole/trimethoprim; *CN*, gentamicin; *LEV*, levofloxacin; *DA*, clindamycin; *E*, erythromycin; *RD*, rifampicin; *TE*, tetracycline.*

## Discussion

Machine-learning techniques can be applied to extract features from bacterial protein sequences (Patel et al., 2017; Huang and Li, 2018; Shao and Liu, 2020; Zhao et al., 2020c). In this study, we successfully use them to distinguish MRSA from MSSA despite their similar sequences. It is reported that MRSA has reached over 60% of all isolated Staphylococcus aureus and the incidence of MRAS has increased to 49% in the United States (Jiang et al., 2020). So, it is very important to identify MRAS from MSSA rapidly. Because traditional assay methods are often time-consuming and with poor sensitivity, our classified recognition method shows its obvious advantages. Here, we have successfully established a machine learning method that based on our develop 188D feature vectors (Li Y. et al., 2019) being able to distinguish MRSA from MSSA. This method shows high specificity and sensitivity, the average discrimination ability reaches more than 90%. Thus, the 188D feature extraction method in this paper could be used as valuable tool for rapid, simple, sensitive and reliable identification of MRSA.

Panton-Valentine leukocidin (*PVL*) is an exotoxin produced by a variety of Staphylococcus aureus isolates that has a strong killing effect on white blood cells (Gauduchon et al., 2001). *PVL*–positive Staphylococcus aureus is highly toxic and is often associated with mild or moderate skin and soft tissue infections (SSTI) and can cause severe invasive infections, including necrotizing pneumonia or invasive bone joint infections (Vandenesch et al., 2012). The prevalence of *PVL* gene in different regions is diverse. In Europe, Glasner et al. (2015) tested 147 *spa* 437-MRSA strains in 11 European countries for *PVL* gene, and the positive rate was as high as 82%. In Asia, the prevalence of *PVL* in CA-MRSA and HA-MRSA was 14.3 and 5.7%, respectively (Song et al., 2012). In Taiwan, the data were also different. The percentage of *PVL*-positive isolates in the study of 253 MRSA strains from blood infection was 11.1% (Wang L. et al., 2010). The detection rate of *PVL* was 45.2% in Sun Yat-sen Memorial hospital of Guangzhou (Xie et al., 2016), and was 47.6% in Hainan’s hospitals in China (Li X. et al., 2019). Consistently in this study, the detection rate of *PVL* was as high as 47.7%. There was no significant difference between CA-MRSA (51.2%) and HA-MRSA (40.9%) groups. The positive rate of *PVL* in this area is relatively high, which suggests that the strong toxicity of MRSA in Quanzhou and the serious invasive infection may result from it. That should be paid more attention by clinicians.

Although *PVL* is usually considered to be a common pathogenic factor for CA-MRSA, some studies have shown that HA-MRSA isolates have a relatively high *PVL* positive rate in some areas (De-Zhi et al., 2011). Zetola et al. (2005) found that the prevalence of *PVL* tends to increase in nosocomial infections. These results suggest that these CA-MRSA may be cloned in hospital environment. Therefore, *PVL* may no longer be a reliable marker for CA-MRSA isolates, but all MRSA may be an important repository of *PVL* virulence factors. This suggests that HA-MRSA with typical CA-MRSA molecular characteristics (*SCCmec* IV and *PVL* positive) has become an important cause of health care related infections.

We found that *CC59-ST59-spa* t437-IV was the predominant clone in Quanzhou. This clone was also one of the most common CA-MRSA strains in East Asia (Song et al., 2012; Chuang and Huang, 2013; Liu G. et al., 2016). In 2007, Tristan and his colleagues reported for the first time the isolates of Staphylococcus aureus *ST59* associated with *spa*-t437 (Anne et al., 2007). Subsequently, a large community and hospital study in Asia described *CC59* as the most popular Complex clone (*CC*). In addition, *ST59-MRSA-t437* was identified as the most prevalent clone between 2004 and 2006 (Song et al., 2012).

In China, *ST59-MRSA-t437-IV* is prevalent among children and adolescents (Li et al., 2013; Ning et al., 2015; Zhen et al., 2017). According to the report by Wang X. et al. (2016), the detection rate of this clone in Shanghai Children’s Medical Center between 2012 and 2013 was 21.3%, while the proportion of *ST59-MRSA-t437-IV* clones detected by Beijing Children’s Hospital in 2016 was as high as 61.7% (Yang et al., 2017). Interestingly, our study found that the proportion of adolescents and children carrying the clone was 36.4% (8/22) in Quanzhou, but the positive rate of the clone was 69.8% (30/43) in adults and significantly higher than that in minors (*p* < 0.05). This clone seems to be more popular in adults. The possible reason is that Quanzhou is located on the southeast coast of China, and the close interaction between local residents and Taiwan, Hong Kong, and Southeast Asian countries have enabled *ST59-MRSA-t437* cloning to have a wide cross-infection among the populations in these areas. Song et al. (2012) also confirmed that community and hospital related MRSA *CC59* strains collected from 8 countries and regions in Asia spread rapidly across national boundaries in both directions. In our study, the clone belonged to CA-MRSA accounted for 67.6% (23/34), HA-MRSA accounted for 32.4% (11/34), and both *SCCmec* type III and IV were present in both community and hospital MRSA infections. This indicated that *CC59-MRSA-t437*-IV can’t be used as a molecular marker for community infection. The difference between CA-MRSA and HA-MRSA has become blurred. More and more CA-MRSA-based clones have successfully invaded into hospital institutions, which has become an important pathogen of infection in hospitals. In many medical centers, they have become a common cause of medical-associated bacteremia (Uhlemann et al., 2014; Chen et al., 2015; Miura et al., 2018).

*CC8-ST239-MRSA*-III is the major HA-MRSA clone in China and some Asian countries, and the corresponding spa typing are mostly t030, t037, and t002 (Chen et al., 2014). The main HA-MRSA clones in Quanzhou were *ST239-MRSA-III/IV-spa* t030. *ST239-MRSA-III-spa* t037 was the most important MRSA clone in Beijing before 2000. Since 2000, *ST239-MRSA-III-spa* t030 has replaced t037 as the main clone (Chen et al., 2010). The most common clones in Shanghai were *ST5-MRSA-II-spa* t002, followed by *ST239-MRSA-III-spa* t037 and *ST239-MRSA-III-spa* t030 (Song et al., 2013). This inconsistent distribution means that the prevalence of MRSA isolates varies considerably even within the same country.

In 2014, China CHINET bacterial resistance surveillance data showed that the resistance rate of vancomycin, teicoplanin and linezolid was zero, ciprofloxacin, sulfamethoxazole/trimethoprim, gentamicin, levofloxacin, clindamycin Erythromycin, and rifampicin were 68.3, 7.0, 62.3, 71.7, 52.9, 77.1, and 47.2%, respectively (Hu et al., 2016). The resistance rate in Quanzhou is generally lower than the national average. *CC59-spat437* is the main MRSA clone in the region, and its resistance rates of ciprofloxacin, levofloxacin, gentamicin and rifampicin are less than 6%, which can be used as the main drug for the treatment of this type of MRSA.

## Data Availability Statement

The original contributions presented in the study are included in the article/supplementary material, further inquiries can be directed to the corresponding author.

## Author Contributions

ZB, MC, and ZL conceived the study and designed the experiments. ZB, KW, DH, and YL collected the strains. ZB, HF, and YY performed the experiments. ZB, MC, QL, KW, JZ, DH, WM, and YL analyzed the data. ZB and ZL wrote the manuscript. All authors reviewed the manuscript.

## Conflict of Interest

The authors declare that the research was conducted in the absence of any commercial or financial relationships that could be construed as a potential conflict of interest.

## Footnotes

1 http://www.uniprot.org/

2 http://cd-hit.org

3 http://www.cs.waikato.ac.nz/ml/weka/

4 https://spa.ridom.de

5 http://saureus.mlst.net
